# Dosimetric comparison of lattice radiotherapy across three modern linear accelerator platforms

**DOI:** 10.1002/pro6.70078

**Published:** 2026-06-05

**Authors:** Shijun Li, Qingliang Pang, Bo Li, Jun Lv, Zhuxin Wei, Dawei Zhang, Wen Qin

**Affiliations:** ^1^ Department of Radiation Oncology The First Affiliated Hospital of Guangxi Medical University Nanning Guangxi China; ^2^ Hengzhou People's Hospital Nanning Guangxi China

**Keywords:** lattice radiotherapy, spatially fractionated radiotherapy, dosimetry

## Abstract

**Background:**

This study aimed to compare the dosimetric characteristics of lattice radiotherapy across different linear accelerator platforms (Versa HD, Halcyon, and NeuRT Aurora) and to evaluate the feasibility of its clinical application.

**Methods:**

This study included ten patients with bulky tumors who were eligible for spatially fractionated radiotherapy, including five pelvic cases and five abdominal cases. For each patient, high‐dose vertices were contoured within the gross tumor volume using a standardized method, followed by three independently designed treatment plans on the Versa HD, Halcyon, and NeuRT Aurora platforms. The prescribed dose was 2400 cGy in three fractions. Dosimetric evaluation of the target volume included vertex coverage, maximum dose, and the peak‐to‐valley dose ratio. In addition, a vertex‐specific dose analysis was performed to quantify the number of vertices with a D95 of less than 2400 cGy. For organs at risk, the following dosimetric parameters were evaluated: the mean dose to the intra‐lattice margin volume, the maximum dose to tissues located more than 2 cm from the vertices, and standard dosimetric parameters for the spinal cord, small bowel, colon, liver, stomach, lungs, and kidneys.

**Results:**

All plans demonstrated the characteristic dosimetric features of lattice radiotherapy by establishing alternating high‐dose peaks and low‐dose valleys within the target volume, while achieving effective protection of surrounding normal tissues without compromising target coverage. Among the three platforms, NeuRT Aurora demonstrated superior performance in dose gradient control and normal tissue protection. The mean peak‐to‐valley ratios were 6.4, 6.9, and 8.9 for the Versa HD, Halcyon, and NeuRT Aurora platforms, respectively. The maximum dose to tissues located more than 2 cm from the vertices was significantly lower in the NeuRT Aurora plans (1158.6 cGy) than in the Versa HD plans (1280.6 cGy) and Halcyon plans (1257.8 cGy). Consistent with this trend, the NeuRT Aurora plans were also associated with a lower mean dose to the intra‐lattice margin volume (792.4 cGy) than the Versa HD (831.4 cGy) and Halcyon (845.7 cGy) plans. For all specific organs at risk, the evaluated dosimetric parameters remained within clinical tolerance limits across the three treatment platforms.

**Conclusions:**

This study systematically evaluated lattice radiotherapy plans across three accelerator platforms. Although differences were observed in peak‐to‐valley ratios and organ‐at‐risk sparing, all plans met clinical requirements, confirming the overall feasibility of each platform.

## Graphical abstract

This study systematically compared the dosimetric characteristics of lattice radiotherapy plans designed on Versa HD, Halcyon, and NeuRT Aurora platforms, providing crucial reference data for future LRT plan design and evaluation

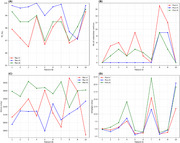

## 1 INTRODUCTION

For patients with locally advanced tumors who are not candidates for surgical resection or local ablation, radiotherapy has emerged as a crucial treatment modality. However, conventional radiotherapy faces substantial challenges when treating large‐volume solid tumors. Achieving tumoricidal doses requires the delivery of high radiation doses, whereas the surrounding normal tissues exhibit limited radiation tolerance, with radiation‐induced toxicity representing the primary dose‐limiting factor.^1^

To overcome this limitation, spatially fractionated radiotherapy (SFRT) has been developed as an innovative technical strategy. This modality intentionally creates a heterogeneous dose distribution within the target volume, characterized by a strategic alternation of high‐dose peaks and low‐dose valleys.^2^, ^3^, ^4^ Its early implementation, known as GRID therapy, used a physical block (e.g., lead or copper) to convert a broad X‐ray field into a pattern of pencil‐beam subfields.^5^, ^6^ Mohiuddin et al.^7^ first demonstrated the successful implementation of megavoltage GRID therapy for bulky tumors, reporting favorable outcomes, including marked symptomatic relief in 93% (20/22) of patients and objective tumor regression in several cases. Subsequent clinical studies consistently established the efficacy and safety of GRID SFRT for bulky tumors.^8^, ^9^, ^10^, ^11^, ^12^ Despite its successes, the two‐dimensional nature of GRID therapy poses significant dosimetric challenges, particularly its inability to achieve conformal dose coverage in deep‐seated or geometrically complex tumors.^13^ Wu et al.^14^ proposed lattice radiotherapy (LRT) in 2010, marking a critical transition of SFRT from a two‐dimensional to a three‐dimensional paradigm. This technique uses advanced treatment planning to create precise three‐dimensional arrays of high‐dose vertices within the tumor volume, thereby enabling superior dose conformity to complex tumor geometries while maintaining the normal tissue–sparing benefits of spatial fractionation.^15^ Growing clinical evidence has demonstrated the successful implementation of LRT across various tumor types, with studies reporting improved local control rates without additional toxicity.^16^, ^17^, ^18^, ^19^, ^20^, ^21^ Peng et al.^16^ reported an 84.2% (16/19) objective response rate with LRT in unresectable head and neck cancer, and Amendola et al.^20^ achieved complete remission in 12 patients with non‐small cell lung cancer. Although numerous publications have documented clinical outcomes, systematic dosimetric investigations remain limited.

The biological mechanisms underlying LRT efficacy, although not yet fully elucidated, are thought to involve several effects. First, the high‐dose vertices directly ablate tumor cells via deterministic radiation effects. Second, the bystander effect may induce cell death in low‐dose regions through intercellular signaling. Furthermore, LRT may potentiate antitumor activity by modulating the tumor microenvironment, including disruption of tumor vasculature and activation of immune responses.^22^, ^23^, ^24^, ^25^, ^26^ These properties render LRT well suited for the treatment of large‐volume tumors.

Advancements in radiation therapy technology have expanded the implementation of LRT to modern platforms, including TomoTherapy, CyberKnife, and particle therapy.^27^, ^28^, ^29^, ^30^ From a cost perspective, LRT based on photon linear accelerator technology offers greater potential for widespread clinical adoption. Photon linear accelerators differ in dose rate, multileaf collimator design, image‐guidance capabilities, and treatment efficiency, all of which directly affect LRT plan quality. In this study, we systematically explored and compared the dosimetric characteristics of LRT plans designed on the Versa HD (Elekta Solutions AB, Stockholm, Sweden), Halcyon (Varian Medical Systems, Palo Alto, CA, USA), and NeuRT Aurora (Neusoft IntelliRay Technology Co., Ltd., Shenyang, China), and evaluated their clinical feasibility.

## 2 METHODS

### 2.1 Configuration of Linear Accelerators

This study employed three photon linear accelerator platforms: the Versa HD, Halcyon, and NeuRT Aurora. The Versa HD was equipped with 80 pairs of 5 mm Agility multileaf collimators and operated with a 6 MV flattening filter–free photon beam at a maximum dose rate of 1400 monitor units (MU) per minute. Treatment planning was performed using Monaco V6.1.1 (Elekta Solutions AB, Stockholm, Sweden), which employs a Monte Carlo algorithm for precise dose calculation. The Halcyon linear accelerator is equipped with a dual‐layer multileaf collimator system and operates at a fixed dose rate of 800 MU/min. The treatment planning system was Eclipse V16.1.0 (Varian Medical Systems, Palo Alto, CA, USA), using the Acuros XB algorithm. Similar to the TomoTherapy system, the NeuRT Aurora linear accelerator delivers radiation with continuous gantry rotation and simultaneous couch translation. It is equipped with a dual‐layer multileaf collimator system and delivers radiation at a dose rate of 1200 MU per minute. The treatment planning system employs DeepPlan V1.4 (Hefei Wisdom Medical Co., Ltd., Hefei, China), which implements a GPU‐accelerated Monte Carlo algorithm for rapid plan optimization. The treatment planning systems of all three platforms were fully commissioned to ensure beam model accuracy.

### 2.2 Patient selection and contouring

Five pelvic tumor cases and five abdominal tumor cases were retrospectively included in this study at The First Affiliated Hospital of Guangxi Medical University between 2024 and 2025. Among the included patients, five were male and five were female. The mean patient age was 53.5 years (range, 30–66 years). A detailed summary of patient characteristics is presented in Table 1. This study was approved by the Institutional Review Board of The First Affiliated Hospital of Guangxi Medical University.

**TABLE 1:** Characteristics of the 10 patients.

ID	Anatomic Site	Sex	Age (years)	GTV Volume (cc)	Lattice Volume (cc)	No. of Vertices	Vertex Volume (cc)	V‐V/V‐L (%)
Case 1	Pelvic	Female	61	814.2	376.3	17	8.6	2.3
Case 2		Female	61	1286.4	605.5	28	14.2	2.3
Case 3		Male	54	1405.4	578.6	29	14.7	2.5
Case 4		Male	54	933.3	379.9	19	9.6	2.5
Case 5		Female	66	1510.1	852.3	40	20.3	2.4
Case 6	Abdominal	Male	30	1348.3	717.1	36	18.5	2.6
Case 7		Male	58	635.2	235.3	12	6.1	2.6
Case 8		Female	48	3083.7	1760.4	84	42.9	2.4
Case 9		Female	49	2307.3	1199.5	56	28.4	2.4
Case 10		Male	54	552.7	180.2	9	4.6	2.6

V‐V/V‐L represents the volume ratio of vertices to the lattice structure.

All patients underwent computed tomography (CT) simulation with a slice thickness of 3 mm using a Philips Big Bore Brilliance CT simulator. The acquired images were transferred to the Monaco treatment planning system for contouring. Following structure delineation, lattice vertices were generated using the smart radiotherapy contouring software, smART Vision Pro V1.3.0 (Shenying MedTech Co., Ltd., Shenzhen, China). Finally, the CT datasets and structure sets were exported to the respective treatment planning systems for plan optimization.

The spatial configuration of lattice vertices is a critical determinant of final plan quality in LRT. Optimal vertex placement must account for three‐dimensional tumor geometry, dose fall‐off gradients, and proximity to organs at risk (OARs). An appropriate vertex arrangement establishes the foundation of LRT by creating high‐dose peaks that deliver therapeutic effects while ensuring that valley doses remain within safe tolerance limits. This study applied the lattice placement method described by Wu et al.^15^ The lattice parameters were configured as follows: a 10 mm inward margin from the gross tumor volume (GTV) boundary, spherical vertices with a diameter of 10 mm, and a center‐to‐center spacing of 30 mm between adjacent vertices. A detailed summary of the lattice structures is presented in Table 1. The GTV volumes ranged from 552.2 to 3083.7 cc, whereas the corresponding lattice volumes ranged from 180.2 to 1760.4 cc. The number of vertices varied from 9 to 84 across cases, with the ratio of total vertex volume to lattice volume maintained between 2.3% and 2.6%.

### 2.3 Planning design

The planning complexity of LRT substantially exceeds that of conventional radiotherapy, primarily because of the need to manage multiple spatially distributed high‐dose vertices. To achieve optimal dose distribution, each LRT plan employed four complete 360‐degree arcs, enabling enhanced dose modulation and precise dose painting throughout the target volume. To account for differences in computational efficiency across planning systems, specific calculation grid settings were implemented: Versa HD plans used a 3 mm optimization grid with 2 mm resolution for final dose calculation; Halcyon plans used a 2.5 mm calculation grid; and NeuRT Aurora plans used a 2 mm grid throughout the optimization process. This adjustment was necessary given the substantial variation in optimization efficiency among platforms and ensured clinically acceptable optimization times.

All treatment plans prescribed a total dose of 2400 cGy delivered in three fractions (800 cGy per fraction). Planning objectives were defined to ensure safe dose delivery while maintaining therapeutic efficacy. Specifically, the maximum dose to any vertex was limited to 3500 cGy, and spatial separation between adjacent high‐dose vertices was ensured by nonoverlapping 2000 cGy isodose lines. Normal tissue protection was achieved by limiting the dose to 1200 cGy at distances greater than 2 cm from the vertex boundary. Additionally, the following regional dose constraints were applied: a mean dose of less than 880 cGy to the region between the GTV and the lattice vertices, and less than 1400 cGy to the region between the lattice volume and the vertices. To further enhance normal tissue sparing, supplemental ring structures were incorporated in all plans to sharpen the peripheral dose gradient. For OARs not immediately adjacent to the target, no additional dose constraints were applied. This study included 30 treatment plans independently designed by two physicists (each with more than two years of experience) and independently reviewed by two senior physicists (each with more than 15 years of experience).

### 2.4 Plan evaluation

A comprehensive dosimetric evaluation framework was implemented to assess the quality of all LRT plans. For the target volume, key evaluation parameters included target coverage (TC), maximum dose (Dmax), and the peak‐to‐valley dose ratio (PVDR). TC was defined as the proportion of vertex volume receiving at least the prescription dose, serving to verify adequate dose delivery to all high‐dose regions. Dmax represented the highest dose delivered to the vertices, which, according to the Mayo Clinic experience, should be less than 150% of the prescription dose.^12^ The PVDR, a key metric of spatial fractionation effectiveness, was calculated as the ratio of the peak dose (represented by the D95 of the vertices) to the valley dose (defined as the mean dose to the 5% of the lattice volume receiving the lowest doses). Owing to computational limitations of the treatment planning systems, dose constraints could not be enforced on individual vertices; therefore, global constraints were applied. Consequently, although the overall TC exceeded 95%, some individual vertices received insufficient dose coverage. Therefore, we quantified the number of underdosed vertices with a D95 of less than the prescription dose. To facilitate comparison of normal tissue protection across cases, two key metrics were analyzed: the mean dose to the intra‐lattice margin volume (Dmargin) and the maximum dose to tissues located more than 2 cm from the vertices (D2cm). For the assessment of specific OARs, SFRT currently lacks well‐established evaluation standards; therefore, existing criteria from stereotactic body radiotherapy were adopted^31^, ^32^. In addition to the maximum dose for all OARs, the following organ‐specific dose‐volume parameters were evaluated: D20cc for the colon, dose‐surface volume (DSV) > 700 cc for the liver (as defined in Table 3), D5cc for the stomach, and D15cc for the kidneys.

## 3 RESULTS

**FIGURE 1:**
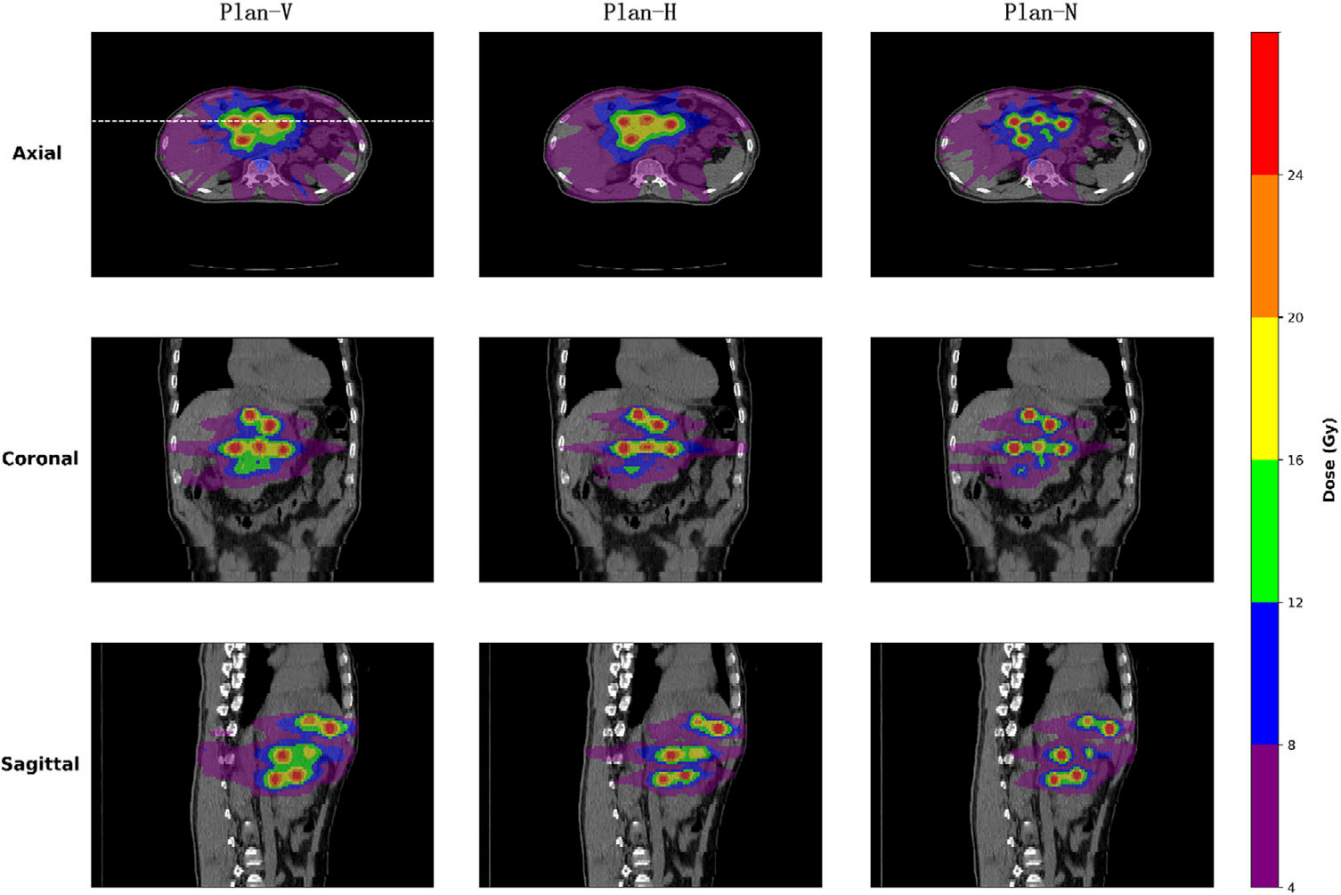
Comparison of dose distributions on computed tomography (CT) images. The images from left to right represent the Versa HD (plan‐V), Halcyon (plan‐H), and NeuRT Aurora (plan‐N) plans, respectively. From top to bottom, the axial, coronal, and sagittal views are displayed. The white dashed lines in the first subfigure indicate the sampling locations of the profiles shown in Figure 2.

**FIGURE 2:**
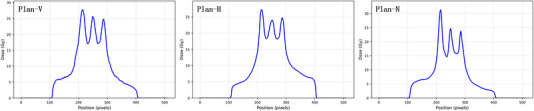
Comparison of lateral dose profiles. The sampling locations for these profiles are indicated by the white dashed lines in the first subfigure of Figure 1.

A comparison of dose distributions across the three accelerator platforms is presented in Figure 1. The layout consists of three columns representing treatment plans generated using the Versa HD (designated as plan‐V), Halcyon (plan‐H), and NeuRT Aurora (plan‐N) systems, respectively. Anatomical orientation is organized in rows displaying axial (top), coronal (middle), and sagittal (bottom) views. All subfigures illustrate radiation dose distributions overlaid on the corresponding CT images, with a unified color scale provided on the right. The dose distribution shown in the figure demonstrates sharp alternation between high‐dose peaks and low‐dose valleys, clearly illustrating the characteristic dosimetric pattern of spatial fractionation. Plan‐N exhibits lower valley doses and steeper dose gradients than the other platforms. Figure 2 presents lateral dose profiles, with sampling locations indicated by white dashed lines in the first subfigure of Figure 1. These profiles allow direct visualization of the periodic alternation between high‐ and low‐dose regions characteristic of the lattice pattern. The variation in peak magnitudes arises because the centers of these vertices are not located within the same CT slice.

**FIGURE 3:**
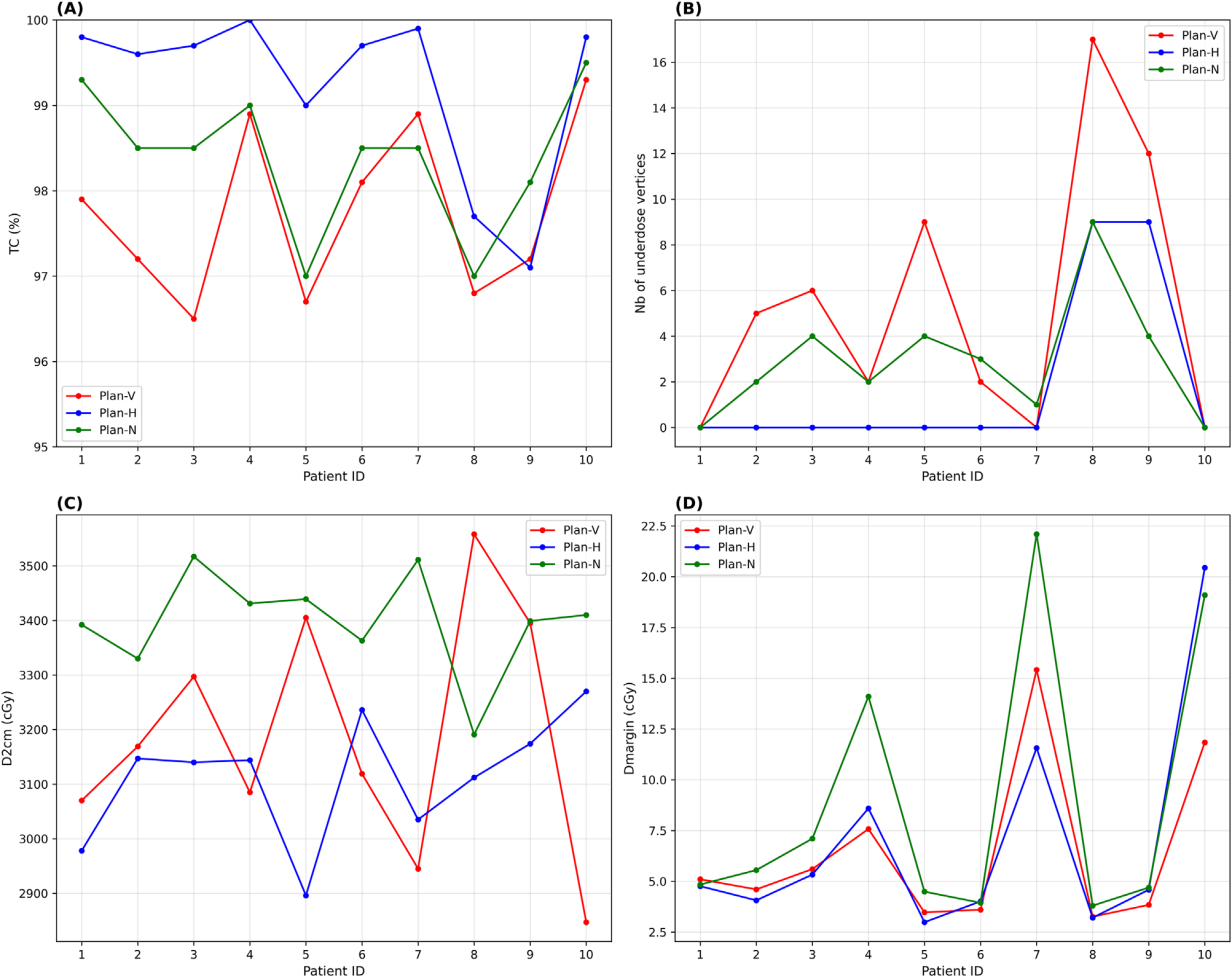
Dosimetric comparison of lattice structures. The results from plan‐V are presented as a red line, whereas plan‐H and plan‐N are shown as blue and green lines, respectively. Subfigure A displays target coverage (TC); Subfigure B presents the number of underdosed vertices; Subfigure C shows the maximum dose (Dmax); and Subfigure D illustrates the peak‐to‐valley dose ratio (PVDR).

When evaluating treatment plan quality, the primary consideration is ensuring adequate dose delivery to the target volume. Therefore, we quantified a comprehensive set of target dosimetric parameters, and the results are presented in Figure 3. Results from plan‐V are shown as a red line, whereas plan‐H and plan‐N are shown as blue and green lines, respectively. Figure 3A presents the comparison of TC, with mean values of 97%, 99%, and 98% for plan‐V, plan‐H, and plan‐N, respectively. All treatment plans demonstrated excellent TC, with values exceeding the 95% clinical threshold. However, in cases with multiple separate targets, the overall TC metric may not accurately reflect the dose coverage of each individual vertex. We quantified the number of vertices failing to achieve D95 > 2400 cGy across all plans, as shown in Figure 3B. Plan‐H demonstrated superior vertex dose coverage, with only two cases presenting underdosed vertices. In contrast, plan‐V exhibited suboptimal coverage in a greater number of cases, along with a higher number of underdosed vertices. In most plan‐N cases, one or two underdosed vertices were observed at the periphery of the lattice volume. The case numbers on the horizontal axis correspond to those listed in Table 1. A clear trend is observed in which cases with higher vertex counts tend to exhibit more underdosed vertices. This correlation may be explained by mutual shielding between vertices, which can block direct radiation to adjacent vertices. Figure 3C displays the maximum dose at the vertices across all plans. Plan‐N achieved higher maximum vertex doses (mean, 3398 cGy) than plan‐V (mean, 3189 cGy) and plan‐H (mean, 3113 cGy). Currently, no standardized upper limits exist for maximum dose in SFRT. All Dmax values in this study remained within 120%–150% of the prescription dose, consistent with the established clinical experience reported by the Mayo Clinic.^12^ The comparison of PVDR across the three platforms is presented in Figure 3D. The PVDR showed considerable variation among cases, with values ranging from 2.9 to 22.1. In general, as the number of vertices increases, planning complexity rises, resulting in a corresponding decrease in the PVDR. Plan‐N demonstrated an advantage in achieving higher PVDR values, with a mean of 8.9 compared with 6.4 for plan‐V and 6.9 for plan‐H.

**FIGURE 4:**
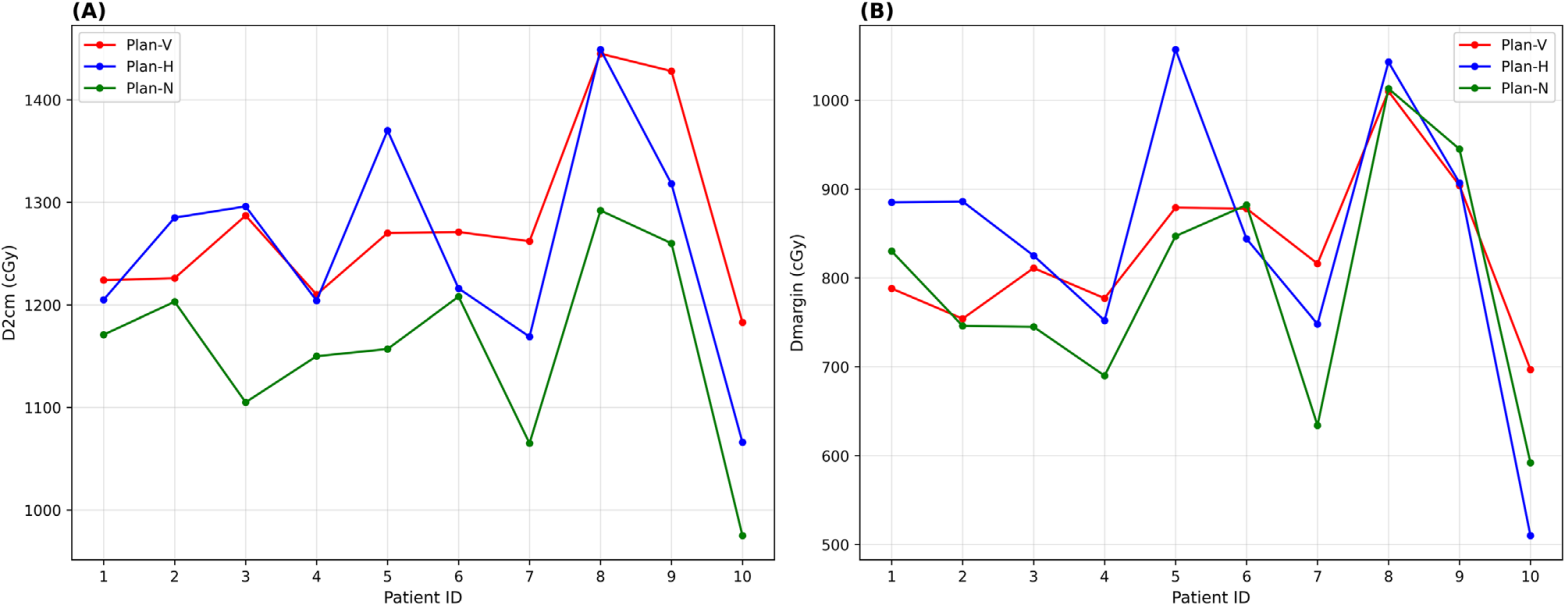
Comparison of geometry‐based evaluation results. The results from plan‐V are presented as a red line, whereas plan‐H and plan‐N are shown as blue and green lines, respectively. Subfigure A displays the maximum dose to tissues outside a 2 cm margin from the vertices (D2cm); Subfigure B presents the mean dose to the intra‐lattice margin volume (Dmargin).

The geometry‐based evaluation results are presented in Figure 4. The mean D2cm values (Figure 4A) were 1280.6 cGy (plan‐V), 1257.8 cGy (plan‐H), and 1158.6 cGy (plan‐N), whereas the mean Dmargin values (Figure 4B) were 831.4 cGy (plan‐V), 845.7 cGy (plan‐H), and 792.4 cGy (plan‐N). These findings indicate that plan‐N provides improved normal tissue protection. The organ‐specific analysis results are summarized in Table 2 (pelvic cases) and Table 3 (abdominal cases). All recorded organ‐at‐risk doses were maintained within accepted stereotactic body radiotherapy tolerance limits, further supporting the clinical feasibility of the generated treatment plans. The Lyman–Kutcher–Burman model^33^ was applied to estimate normal tissue complication probability (NTCP) for the liver, kidneys, and spinal cord. The results showed that NTCP values for all evaluated plans were below 0.001%. Taking Case 6 (plan‐V) as an example, the calculated NTCP values were 2.213 × 10^−^^4^% for the liver, 1.128 × 10^−7^% for the kidneys, and 1.128 × 10^−7^% for the spinal cord, indicating that the predicted risk of radiation‐induced complications was clinically negligible. These exceptionally low NTCP values can be attributed to the steep dose fall‐off gradient inherent to LRT and the inward margin, which ensure that high‐dose vertices remain at a safe distance from critical organs.

**TABLE 2:** Dosimetric Comparison of Organs at Risk Across Three Accelerator Platforms in Pelvic Cases.

	Spinal Cord	Small Bowel	Colon	
	Dmax (cGy)	Dmax (cGy)	D20cc (cGy)	Dmax (cGy)
Case 1				
Plan‐V	234	1247	695	1031
Plan‐H	213	1272	622	1003
Plan‐N	266	1272	670	1138
Case 2				
Plan‐V	447	1120	748	1103
Plan‐H	392	1093	741	1133
Plan‐N	523	1276	770	1137
Case 3				
Plan‐V	746	1242	898	1243
Plan‐H	734	1267	855	1226
Plan‐N	838	1187	838	1119
Case 4				
Plan‐V	612	1204	963	1170
Plan‐H	587	1167	745	1110
Plan‐N	551	1181	870	1261
Case 5				
Plan‐V	795	1362	618	1206
Plan‐H	628	1463	718	1240
Plan‐N	849	1218	560	1096

**TABLE 3:** Dosimetric Comparison of Organs at Risk Across Three Accelerator Platforms in Abdominal Cases.

	Liver‐GTV		Stomach		Lungs	Kidney		Spinal Cord	Colon	
	DSV>700cc	Dmax	D5cc	Dmax	Dmax	D15cc	Dmax	Dmax	D20cc	Dmax
Case 6										
Plan‐V	700	1554	452	648	2830	696	1317	790	271	1226
Plan‐H	717	1590	254	468	2728	457	1024	523	241	1196
Plan‐N	753	1793	299	423	3031	637	1132	731	182	1062
Case 7										
Plan‐V	336	1497	897	1239	737	540	860	770	614	1066
Plan‐H	287	1466	891	1207	440	515	790	508	615	930
Plan‐N	251	1294	823	1088	452	416	743	672	482	961
Case 8										
Plan‐V	153	1392	518	775	49	594	967	1043	1170	1528
Plan‐H	146	1406	818	1129	70	859	1285	1045	1119	1501
Plan‐N	59	1364	372	602	25	559	906	896	1093	1376
Case 9										
Plan‐V	90	1488	666	982	53	526	1257	823	948	1367
Plan‐H	69	1564	659	889	37	460	1332	724	936	1471
Plan‐N	44	1198	464	729	17	518	1167	980	1016	1515
Case 10										
Plan‐V	246	1486	215	291	1114	368	538	358	278	550
Plan‐H	149	1206	117	167	1017	285	498	200	129	329
Plan‐N	185	1371	157	265	1027	260	439	279	193	376

*Note*: DSV>700cc refers to the dose to the hottest organ volume after excluding 700 cc (i.e., D[Vorgan ‐ 700cc]). All values are reported in cGy. DSV, dose‐surface volume

## 4 DISCUSSION AND CONCLUSIONS

As an innovative SFRT technique, LRT has emerged as an important treatment option for bulky tumors because of its distinctive three‐dimensional distribution of high‐dose vertices. This dose configuration exhibits a pronounced peak‐to‐valley gradient, in which high‐dose vertices achieve concentrated dose deposition while low‐dose regions maintain a minimal radiation background within the treatment volume. Through this spatially precise modulation, LRT enables nonuniform dose painting within the target, thereby leveraging the physical advantages of SFRT. Given this unique dose distribution, precise alignment of high‐dose vertices with the intended tumor location is critically important. At our center, several LRT cases have been delivered using the Versa HD platform. In our clinical workflow, cone‐beam computed tomography (CBCT)‐based image‐guided radiotherapy is performed before each LRT fraction using grayscale registration. It is important to note that, because vertices are virtual volumes defined on the planning CT and lack corresponding anatomical structures on CBCT, image registration is based primarily on the bulk tumor rather than on individual vertices. For abdominal cases, abdominal compression plates are used to limit respiratory‐induced organ motion and minimize target displacement, thereby preventing dose “blurring” of the peak‐to‐valley distribution caused by organ movement. Regarding interfraction tumor shrinkage, the most commonly used fractionation scheme in our LRT clinical protocol consists of three fractions. Based on our experience, significant interfraction tumor regression is rarely observed on CBCT images during this short treatment course. Nevertheless, to address potential anatomical changes, all patients undergo a repeat CT scan after the third fraction, followed by recontouring and replanning before subsequent treatments. This approach helps mitigate the impact of tumor regression on dose delivery accuracy. However, we acknowledge that a three‐fraction interval before replanning may not capture earlier geometric changes. To address this limitation, emerging online adaptive radiotherapy offers a promising solution by enabling real‐time plan adaptation based on daily anatomical variations. This strategy has the potential to further enhance the therapeutic ratio of LRT by maintaining dosimetric accuracy while minimizing geographic miss and normal tissue exposure.

LRT is not only a technique for physical dose delivery but also a potent trigger of multifaceted biological responses. The biological mechanisms of LRT extend beyond direct cytotoxicity. High‐dose vertices induce DNA damage and promote the release of proapoptotic factors, thereby triggering bystander cell death in nonirradiated regions and suppressing tumor regeneration.^34^ Concurrently, LRT promotes immune activation by facilitating tumor antigen release and the secretion of damage‐associated molecular patterns, thereby enhancing CD8‐positive T‐cell infiltration and reversing immunosuppression within the tumor microenvironment.^35^ Additionally, low‐dose valley regions contribute to vascular normalization by promoting endothelial repair and downregulating hypoxia‐inducible factor‐1α expression, which alleviates hypoxia and improves tumor oxygenation.^36^

As understanding of immunomodulation and the bystander effect deepens, LRT is gaining increasing clinical interest. Through systematic dosimetric analysis, this study comprehensively evaluates, for the first time, the clinical potential of the Versa HD, Halcyon, and NeuRT Aurora platforms in LRT and provides a reference for future LRT plan design and evaluation. Despite variations in plan quality, the overall results demonstrate consistent achievement of approximately 98% TC, with valley doses maintained at approximately 20% of peak doses. The maximum dose at 2 cm beyond the vertices was limited to approximately 55% of the prescription dose, whereas the mean dose to the intra‐lattice margin volume remained at approximately 35% of the prescription dose. Comparative analysis demonstrated improved performance of the NeuRT Aurora platform in achieving sharper dose gradients and enhanced normal tissue protection. The NeuRT Aurora platform achieves enhanced modulation capability by integrating continuous couch translation with gantry rotation, thereby enabling access to a broader range of beam angles than the other platforms. In a similar study, Sheikh et al.^27^ employed two virtual GRID phantoms to perform a systematic dosimetric comparison of three‐dimensional conformal radiotherapy, volumetric modulated arc therapy (VMAT), and tomotherapy‐based techniques. Their work confirmed that all three modalities can achieve spatially fractionated dose distributions characterized by alternating high‐ and low‐dose regions. Grams et al.^13^ performed a comparative dosimetric analysis of VMAT‐based LRT, brass grid therapy, and proton grid therapy and provided recommendations regarding their potential clinical applications.

For cases with higher vertex counts (e.g., Case 5 with 84 vertices and Case 6 with 79 vertices), we observed that although overall TC exceeded 95%, a substantial number of individual vertices remained underdosed. This phenomenon can be attributed to inherent limitations in current treatment planning system optimization algorithms. Once optimization meets the preset overall TC criteria, priority may shift from individual vertex dose coverage to normal tissue sparing, thereby leading to underdosing of multiple vertices. Furthermore, the finite modulation capability of the multileaf collimator may physically limit direct beam exposure to some vertices. We also observed that cases with higher vertex counts typically exhibited lower peak‐to‐valley dose ratios. This finding likely results from dose interference between adjacent high‐dose regions, which reduces the peak‐to‐valley dose differential and produces a more homogenized dose distribution.

There are several limitations to this study. Vertex placement was guided by geometric rather than biological imaging, which may have resulted in high‐dose regions missing biologically active tumor areas or being positioned within necrotic tissue. The primary objective of this study was to compare the dosimetric performance of LRT plans designed on three different accelerator platforms. To ensure consistency in this comparative analysis, a standardized and fixed set of lattice parameters was employed. In clinical practice, parameter selection should be individualized according to patient‐specific anatomy, tumor characteristics, and proximity to OARs, and it relies heavily on the experience and clinical judgment of physicians and physicists. The prescribed dose was 2400 cGy delivered in three fractions; however, optimal dosing likely requires individualization based on patient‐specific factors. Furthermore, whether a higher PVDR translates into meaningful clinical benefit remains uncertain, as current evidence does not consistently demonstrate an association with improved treatment outcomes. To date, the biological mechanisms underlying SFRT remain incompletely elucidated, and a standardized plan evaluation framework has not been established. The absence of such standards results in substantial variation in the selection and calculation of dosimetric parameters across institutions. The therapeutic efficacy of LRT‐induced immune activation and bystander effects also warrants further investigation and clinical validation in larger patient cohorts.

To date, the biological mechanisms underlying SFRT remain incompletely elucidated, and a standardized plan evaluation framework has not been established. The absence of such standards results in substantial variation in the selection and calculation of dosimetric parameters across institutions. Furthermore, the clinical significance of a higher PVDR remains uncertain, as it has not been consistently associated with superior clinical outcomes. Ultimately, clinical decision‐making relies on observable endpoints, including significant tumor reduction, effective symptom relief, acceptable toxicity, and sustained local control. It should be noted that the plans in this study were not delivered clinically; therefore, their therapeutic efficacy requires validation in future clinical applications.

Future research should move beyond current semi‐automated vertex placement toward fully intelligent, biology‐driven treatment planning and automation for LRT. Although existing tools have simplified the planning process, optimal selection of vertex parameters—such as size, spacing, and weighting—remains an area requiring further investigation. Integrating multimodal imaging biomarkers and biological effect models into the optimization engine could enable personalized vertex configuration that balances dosimetric gradients with anticipated biological responses. Furthermore, development of automated plan quality assurance frameworks and knowledge‐based planning systems will facilitate standardized LRT delivery across institutions, thereby supporting large‐scale clinical trials and broader clinical implementation.

In summary, treatment plans designed on all three linear accelerator platforms successfully demonstrated the characteristic lattice features of spatially fractionated dose distributions. Comparatively, the NeuRT Aurora platform exhibited advantages in dose gradient control and normal tissue protection.

## CONFLICT OF INTEREST STATEMENT

The authors declare no competing interests.

## ETHICS STATEMENT

This retrospective study was conducted using historical patient computed tomography (CT) images obtained from our institution. The study protocol was approved by the Ethics Committee of The First Affiliated Hospital of Guangxi Medical University (Approval No. 2025‐E0888) and was conducted in accordance with the ethical standards of the 1964 Declaration of Helsinki and its subsequent amendments. The requirement for informed consent was waived by the Institutional Review Board because of the retrospective design of the study.

## Data Availability

Due to ethical and privacy restrictions, the raw data supporting the findings of this study cannot be made publicly available. Data may be available from the corresponding author upon reasonable request, subject to institutional approval.
